# Successful Management of a Penetrating Cardiac Injury by Date Palm Thorn: A Case Report

**DOI:** 10.7759/cureus.79118

**Published:** 2025-02-16

**Authors:** Nada A Aljassim, Sami Al-Ahdal, Omar Altamimi, Sami Alahmari, Nabeel Almashraki

**Affiliations:** 1 Pediatric Intensive Care Units, King Fahad Medical City, Riyadh, SAU; 2 Cardiac Surgery, King Fahad Medical City, Riyadh, SAU; 3 Pediatric Cadiology, King Fahad Medical City, Riyadh, SAU; 4 Pediatric Critical Care, King Fahad Medical City, Riyadh, SAU

**Keywords:** cardiac injury, chest, palm tree, penetrating, thorn, trauma

## Abstract

Penetrating cardiac injury in children is a life-threatening emergency. The initial reaction of the witness and medical team management plays a crucial role in survival. We are reporting a five-year-old girl who had a penetrating cardiac injury after an accidental fall on a sharp date tree thorn, witnessed by her parents. They took their child immediately to the hospital without manipulating the thorn. The initial workup included an X-ray, echocardiography, and computed tomography (CT) of the chest, revealing that the thorn transfixed the heart from the anterior chest wall to the posterior mediastinum. The team controlled the child’s pain with analgesia and stabilized the vital signs awaiting transport to a cardiac center. In our center, the child was evaluated by a multidisciplinary team and then was taken to the OR. The surgical team extracted the thorn successfully on cardiopulmonary bypass and excluded any secondary injury. The child had an uneventful hospital course.

Penetrating cardiac injuries by sharp objects from trees could happen to children. The outcome of cardiac injury depends on many factors, such as the extent of the injury, hemodynamic stability on arrival, and pericardial tamponade. The initial management, transportation by a skilled team, and planned surgical intervention are crucial to maintaining the patient’s life with penetrating cardiac injuries.

In conclusion, deep-seated penetrating cardiac injury in children is a potential cause of death. Early recognition, management, and surgical intervention are crucial for favorable outcomes.

## Introduction

Penetrating cardiac injury in children can be fatal [1]. The reported incidence of penetrating cardiac injuries is about 7%, with a low in-hospital survival (less than 30%) [1]. Survival depends on many factors, including the mechanism of injury, the extent of cardiac injury, the location of significant injury, the presence of tamponade, the patient’s vital signs and hemodynamic response on one side, and the initial patient’s management on the other [2,3]. Few children were reported to have penetrating cardiac injuries, including thorns [4-6]. We report a child who had an accidental penetrating trauma to her chest with a date palm thorn. It resulted in a deeply seated thorn that penetrated the heart and transfixed the heart between the anterior chest wall and the posterior mediastinum. She had initial stabilization and then required air transportation to a facility with pediatric cardiac surgery that was 1000 kilometers away. Our case report differs from previous cases in two ways: first, we had a successful outcome even though the injury was severe, and the patient arrived 12 hours after the accident. Second, it proved that teamwork, good communication, and a well-planned surgical procedure could dramatically change the outcome.

## Case presentation

**Figure 1:**
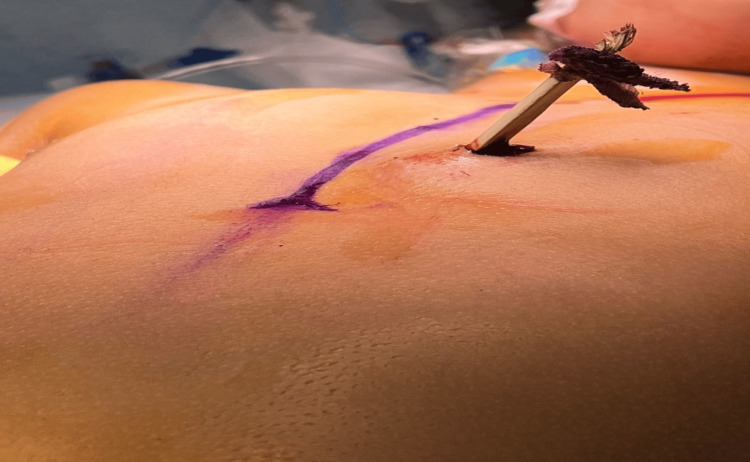
The thorn penetrated the child's chest.

A five-year-old girl living in a rural area was taken to a nearby emergency department (ER) by her parents after falling on a palm tree thorn that penetrated her chest (Figure 1).

**Figure 2:**
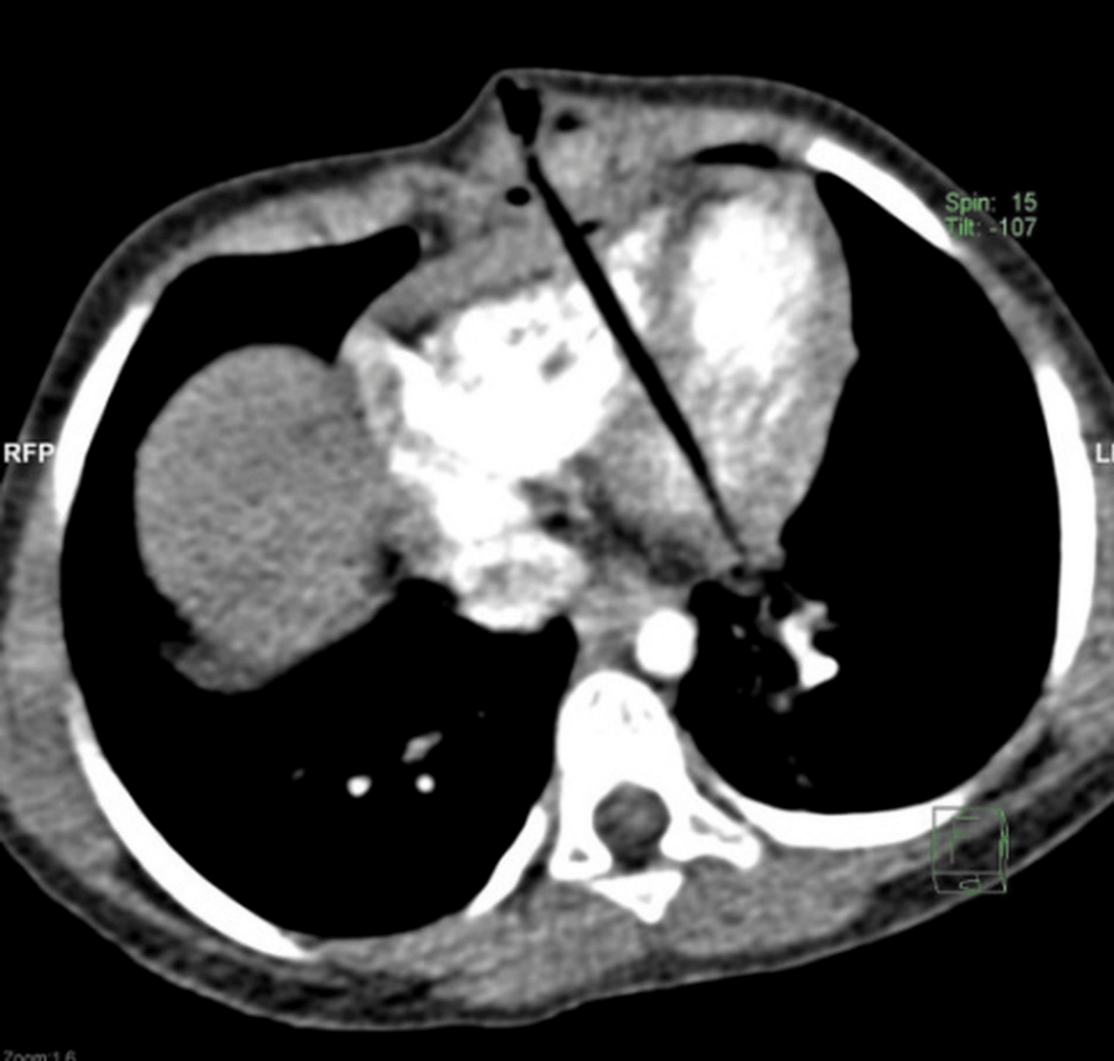
CT chest shows the thorn through the right ventricle (RV), interventricular septum (IVS) and distal tip penetrating the left ventricle (LV).

She was conscious but in pain. Her blood pressure was 75/52 mmHg, her respiratory rate was 45/min, and her oxygen saturation was 95% on supplemental oxygen via face mask. She had a clinical picture of uncompensated shock in the form of weak peripheral pulses and delayed capillary refill. Their initial management included fluid resuscitation, normal spontaneous breathing, and oxygenation. Central venous access was inserted, which showed a central venous pressure of 6 mmHg. Chest X-ray showed average lung volume and no pneumothorax, pleural effusion, or cardiomegaly. The echocardiography revealed the penetration of the thorn through the anterior right ventricle (RV) wall, the interventricular septum (IVS) and crossing the posterior left ventricle (LV) wall. Other cardiac structures were intact, with minimal pericardial effusion and normal LV systolic and diastolic function. A CT chest (Figure 2) showed a linear foreign body penetrating the anterior chest wall, pericardium, RV, IVS, posterior LV wall, and possible posterior pericardium. Its distal tip seems to be at the LV, surrounded by small focal hypodensity. A mild anterior dense pericardial effusion is noted, suggesting pericardial hemorrhage. There is a hematoma on the anterior chest wall at the site of penetration but no pleural effusion or pneumothorax. Major thoracic vascular structures are grossly unremarkable. The major tracheobronchial tree is patent. The child was referred to us as life-saving for further intervention and management.

A high-risk transportation was arranged to our cardiac center with precautions for stabilization and avoiding object movement through the protruding piece outside the chest. Upon arrival at our hospital, she was handled by a multidisciplinary team, including ER staff, a pediatric cardiac surgeon, a pediatric interventional cardiologist, a pediatric cardiac anesthesiologist, and pediatric cardiac intensivists. The patient was conscious, tachypneic with shallow breathing, on morphine infusion for pain. The heart rate was 116 beats per minute, and blood pressure was 82/50 mmHg. Oxygen (O2) saturation was 92% on O2 via a face mask of 8 liters per minute. There was no active obvious bleeding. The child was given broad-spectrum antibiotics (Tazocin at 300 mg/kg/day and vancomycin at 30 mg/kg/day) and antifungal therapy as prophylaxis (fluconazole 5 mg/kg/day). 

**Figure 3:**
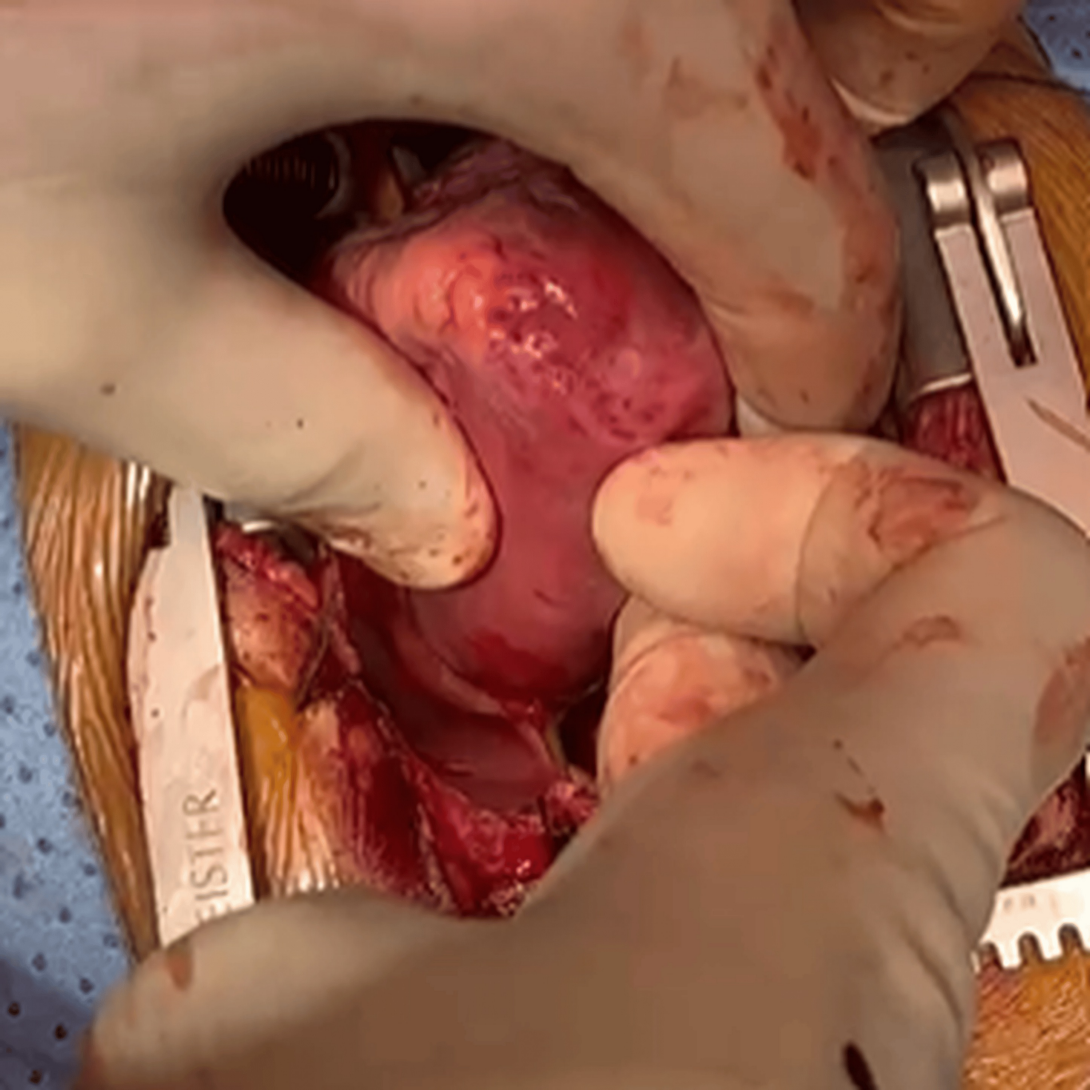
Posterior pericardium penetration point.

We repeated the computed tomography scan as an emergency and then moved to the operation room (OR) for the thorn extraction with a subspecilized team including cardiovascular surgery, cardiothoracic surgery and the pediatric interventional cardiology team for hybrid intervention if required like secondary IVS defect closure. A rapid sequence intubation was performed smoothly on the child and a transesophageal echocardiography (TEE) was performed before initiating the cardiopulmonary bypass (CPB) to assess the magnitude of LV penetration, which showed that the thorn was protruding posteriorly from LV. Then, femoral-femoral cannulations were established for CPB initiation especially with the presence of the thorn close to the sternum. No cross-clamp to the aorta or cardioplegia was given. Then, the sternum was opened and the thorn was cut between the pericardium and chest wall to remove the external part. Then, the pericardium, which had a blood collection around it, was opened. A right atrial cannulation was added to secure adequate venous drainage, and the pericardium was released from the thorn. The thorn penetrated the free wall segment of the RV, IVS and projected from the posterior wall of the LV, to the posterior wall of the pericardium for about 0.5 cm (Figure 3) touching the adventita of the descending aorta.

**Figure 4:**
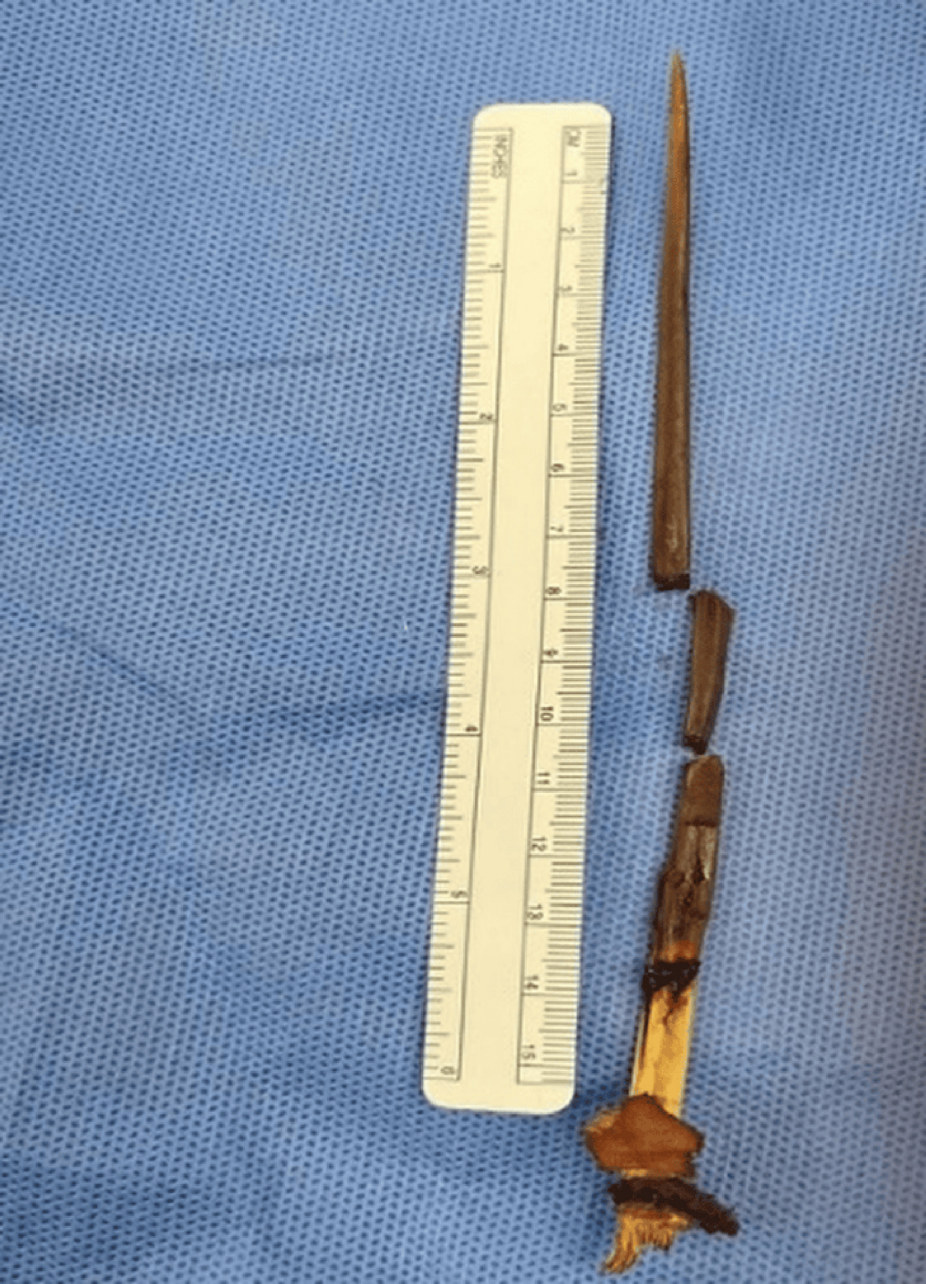
Full length of the thorn.

The LV posterior wall was repaired after pulling the distal tip of the thorn to the LV cavity by an interrupted pledgetted 4/0 polypropylene sutures. Then, the whole thorn was pulled slowly; it was almost 13 cm deep (Figure 4), and the RV injury site was also repaired. There was no coronary injury. No blood products were required through the surgery except at the time of CPB priming.

Post-procedure, the LV filling was resumed, and repeated TEE revealed good ejection without residual IVS defect and without valvular regurgitation. The child was weaned from CPB and the chest was closed uneventfully, then shifted to the pediatric cardiac intensive care unit in stable condition. On the following day, the child was extubated and transferred to the cardiac ward in good condition, with unremarkable post-operative echocardiography findings.

Later, the child attended school with good activity, and the family or child didn’t express any post-traumatic psychotic impact.

## Discussion

Pediatric cardiac injury is associated with high mortality regardless of the child’s age, reaching up to 40% [7]. However, early identification and surgical intervention are associated with favorable outcomes [8]. Date palm thorn-related cardiac injuries are rare in the Middle East region, although rarely reported. A case reported by De Decker et al. was presented as a pulsatile pericardial mass secondary to penetrating thorn injury, with the sub-acute presentation [4]. The injury was limited to the right ventricle with fistula formation. In this case report, the presentation was acute, and the injury's extent crossed many heart structures and involved mainly the LV. Although the outcome of penetrating cardiac injury depends on the mechanism of injury and extent, early recognition and initial management are crucial steps [1-3,6,8-10]. For instance, the initial measures taken at the scene by the witness and medical team before referring the patient to a specialized cardiac center are fundamental to saving the patient, including the transport team’s experience in handling the situation to ensure safety, which was done very well in this case. Also, a multi-disciplinary approach with adequate workup to evaluate the trauma extent and interventional plan of care are crucial to managing any cardiac and collateral damage to the penetrating cardiac injury [11]. Fortunately, the child was saved despite the difficulties encountered in transferring her quickly for urgent intervention at the cardiac center. The case shed light on the importance of having clinical care delivery at each low-resource region and facilitation to reach higher centers with sub-specialized care including pediatric cardiac surgery, cardiothoracic surgery and interventional cardiology.

## Conclusions

Deep-seated penetrating cardiac injury in children is associated with high risk of death. Early recognition and management till surgical intervention is crucial. The medical-surgical management requires teamwork and specialized care to create the best interventional care plan.
